# Guarding minds: a narrative review on how n-acetylcyteine and ketones could shield sensitive patients from antibiotic neurotoxicity

**DOI:** 10.3389/fphar.2025.1613152

**Published:** 2025-06-13

**Authors:** Astrid Lounici

**Affiliations:** University Psychiatric Clinic Basel, Basel, Switzerland

**Keywords:** antibiotic neuropsychiatric toxicity, neurological adverse effects, psychiatric side effects, mitochondrial dysfunction, oxidative stress, antioxidants (N-acetylcysteine -NAC), ketogenic diet and ketones, neuroprotective strategies

## Abstract

**Background:**

Antibiotics, indispensable in combating infectious diseases and extending life expectancy, are among the most commonly prescribed medications globally. However, neurotoxicity, encompassing neurological and psychiatric adverse effects, is an underrecognized phenomenon associated with all major classes of antibiotics. Certain antibiotics, such as fluoroquinolones, carry risks of permanent damage, including central and peripheral nervous system injury and mitochondrial dysfunction. Sensitive populations, such as the elderly and individuals with compromised organ function or genetic predispositions, are particularly vulnerable.

**Aims:**

To review evidence on the neurological and psychiatric side effects of antibiotics and evaluate potential neuroprotective strategies like N-acetylcysteine (NAC) and ketone bodies.

**Method:**

Narrative review of preclinical and clinical studies, clnical case reports and epidemiological data.

**Results:**

Neurological and psychiatric side effects are rare, but they can be devastating. NAC shows promise in preclinical studies for mitigating oxidative stress and cellular damage. Ketones, through ketogenic diets or exogenous supplementation, may provide neuroprotection *via* enhanced mitochondrial function and anti-oxidative and anti-inflammatory effects.

**Conclusion:**

While initial findings are promising, further research is required to validate the clinical efficacy of these protective agents. Improved understanding of antibiotic neurotoxicity and potential mitigation strategies could lead to safer prescribing practices, particularly for vulnerable populations, balancing risk mitigation with the essential benefits of antibiotics.

## Introduction

Antibiotics are among the most prescribed medication (The Top 300 of 2021, n.d.) and prescriptions had a special increase during the COVID-19 pandemic (Nandi et al., 2023). According to Centers for Disease Control and Prevention (CDC) in US healthcare professionals prescribed 236.4 million antibiotic prescriptions—equivalent to 709 antibiotic prescriptions per 1,000 persons in the year 2022. Since their first discovery in 1910, they have contributed to the combat of infectious diseases, making it possible to save lives and increase life expectancy by 23 years (Hutchings et al., 2019). Antibiotics are indispensable in modern medicine. They not only combat community-acquired infections but also enable complex cancer chemotherapies, protecting patients who would otherwise succumb to the immunosuppressive effects of the treatment. Additionally, antibiotics are crucial for surgical procedures, such as complex bowel surgeries, which would frequently result in deadly infections without their protection.

Despite their great contribution to healthcare worldwide, all classes of antibiotics can have a wide range of neurotoxic effects (Grill and Maganti, 2011; Rezaei et al., 2018). Neurological and psychiatric side effects are generally estimated to be rare (Shehab et al., 2008), although they are often underrecognized (Lacroix et al., 2024). Allergic reactions are the most common adverse events, accounting for 75% of emergency department visits (Geller et al., 2018), and neurological side effects are often temporary and subside when the medication is stopped (Lacroix et al., 2024). However, some antibiotics are known to sometimes cause long-term damage to the central and/or peripheral nervous system. For instance, fluoroquinolones carry an FDA black box warning due to their potential to cause permanent nerve damage (U.S. Food and Drug Administration, 2016). Additionally, nitrofurantoin, though rarely, can lead to irreversible damage to the peripheral nervous system even after short-term use (Yiannikas et al., 1981). Metronidazole, which can cause dysarthria, gait instability, limb dyscoordination, and altered mental status, has been reported to cause lesions visible on MRI, which in most cases, but not all, resolve after discontinuation of the drug. Encephalopathy by antibiotics mostly happens with impaired hepatic or renal function, but it can also happen with intact function (Sørensen et al., 2020). While most neurological side effects resolve after discontinuing the antibiotic, the potential for serious long-term effects in a subset of sensitive individuals remains a concern. Due to insufficient support, self-help groups such as Floxie Hope have emerged Floxie Hope, 2022). Some antibiotics can potentially cause depression (Hao et al., 2020) and neurological and psychiatric side effects can lead to life threatening situations with prolonged hospitalization and even disability (Bennett et al., 2022; Lacroix et al., 2019; Wilcox et al., 2020; Xiao and Huang, 2021).

### The difficulty of attributing side effects to antibiotics

Although antibiotic side effects are more common with higher dosages, they can also be idiosyncratic (Andrade and Tulkens, 2011). While older populations and individuals with decreased kidney or liver function seem to be more frequently affected, all age groups, including previously healthy individuals, can experience neurotoxic effects even with standard dosages and short-term use (Kaur et al., 2016). Side effects often occur only after the cessation of the antibiotic. For example, hepatic dysfunction that can become chronic after amoxicillin/clavulanate or tetracycline therapy may develop almost immediately or several months after the cessation of therapy (Andrade and Tulkens, 2011). Nitrofurantoin peripheral nerve damage has been reported to happen 1 week after cessation of the medicament (Yiannikas et al., 1981). Also, psychiatric symptoms can occur long after cessation of an antibiotic, making it difficult to detect as a side effect (Kaur et al., 2016).

### Tissue damage by antibiotics

The neurological and psychiatric side effects of antibiotics have the potential to become chronic as antibiotics have the ability to leave alterations in tissue. Well-known permanent damage is associated with nitrofurantoin, which, in addition to causing peripheral nerve damage, can lead to lung tissue damage after long term use with an insidious onset (Wilkin et al., 2009) or macrolides, which can lead to heart cell fibrosis as observed in a rat model. (Farag et al., 2021).

The brains of mice treated with clinically relevant doses of ciprofloxacin showed significantly higher levels of apoptosis and brain tissue necrosis compared with controls. Also significantly higher expression in levels of the HMGB-1 protein, that is involved in inflammation, was found in the mice treated with ciprofloxacin (Kaur et al., 2016).

In this narrative review, we will describe the neurological and psychiatric side effects of various antibiotics, shed light among others on mitochondrial toxicity as possible mechanism, and summarize findings from epidemiological studies that specifically examine antibiotic use and infections.

The final part will discuss the agents n-acetyl cysteine and ketones that could potentially be used as protection against neurotoxicity.

## Methods

I conducted a literature search using Google Scholar and PubMed. The following search terms were used: *antibiotics and tissue toxicity/neurotoxicity*, *antibiotics and psychiatry*, *use of antibiotics and risk of psychiatric disease*, *antibiotics and mitochondrial dysfunction/mitochondrial toxicity*, *mitochondrial dysfunction and inflammation*, *antibiotic toxicity and N-acetylcysteine (NAC)*, and *ketogenic diet/ketones neuroprotection.*

Whenever possible, I prioritized human studies. In the absence of human data, I included animal studies, and in one case, *in vitro* data.

To complement the database searches, I also manually reviewed the reference lists of selected studies and relevant review articles. This step helped identify key publications that may not have appeared in the database search, contributing to a more comprehensive overview of the literature.

## Results

### Psychiatric side effects of antibiotics

Given the widespread use of antibiotics, psychiatric adverse events of antibiotics are rare but are not always recognized. In the elderly, for example, cognitive side effects are often missed or misdiagnosed (Farrell and Ganzini, 1995) or unspecified side effects such as sleep disturbances are not recognized (Neufeld et al., 2017).

The incidence of psychiatric side effects for fluoroquinolones seems to be low. In 2019, there were 13.7 million fluoroquinolone prescriptions in the USA, and the reported psychiatric side effects were 1,000 per year among those prescriptions. This is a prevalence of seven per 100,000 (Umarje et al., 2021).

Nevertheless, fluoroquinolone toxicity can be very serious and long lasting. In UK there is a government safety update from 2024 that fluoroquinolones should be prescribed only when other antibiotics fail (GOV.UK).

For fluoroquinolones the following psychiatric side effects were described: abnormal dreams, nightmares, acute psychosis, agitation, aggression, anxiety, confusional state, delirium, delusion, depression, euphoric mood, hallucinations, hostility, insomnia, irritability, mania, manic depression, mood swings, nervousness, panic attack, paranoia, personality disorder, psychotic disorder, sleep disorder, somatic delusion and suicidal ideation (Tomé and Filipe, 2011).

Neufeld et al. looked for psychiatric side effects after antibiotic treatment for *Helicobacter pylori* infection. This infection is typically treated with a so-called triple therapy, which includes a proton pump inhibitor, clarithromycin, along with amoxicillin or metronidazole. They conducted a literature search and found that the most common symptoms were gastrointestinal, followed by neurological adverse events, while psychiatric adverse events were less common (Neufeld et al., 2017).

Abusafiyah and Soulen searched the FDA Adverse Event Reporting System (FAERS) database for a timeframe of 10 years and found the following numbers of psychiatric side effects: for Beta-Lactam antibiotics, 2,217 severe psychiatric side effects were reported; for SMX/TMP, 2,761; for Vancomycin, 581; for Gentamycin, 90; and for Metronidazole, 1,730. In all those classes, deaths were also reported, but the authors did not specify if those deaths were due to psychiatric side effects or other causes (Abusafiyah and Soulen, 2023).

Quinolones have been associated with suicide. Samide et al. looked at the WHO database for individual case safety reports (“VigiBase”) between 1970 and 2015 and found 608 cases of suicidal behavior with 97 cases of completed suicide. There was an increased odds ratio (2.78) compared to other antibiotics (Samyde et al., 2016).

Mania is a rare problem associated with antibiotics, and the term “antibiomania” has been coined (Abouesh et al., 2002). Lambrichts et al. who conducted a literature review found only 46 cases. The authors state that only in 39% of cases did the symptoms subside without adding antimanic medication (Lambrichts et al., 2017).

Delirium after antibiotics can happen especially often in the elderly with urinary tract infections (UTI’s) because of the infection itself (Dutta et al., 2022). Nether the less, psychosis was associated with antibiotic treatment of UTI. Fifteen cases were identified in a literature search, with 60% being highly suggestive of the antibiotic being causative, including three cases where there was a recurrence of psychosis after the same antibiotic was given again. The psychosis occurred in individuals aged 18–88 with a mean age of 50.8. The duration of psychosis onset was 4.8 days and only half of the cases required treatment with antipsychotics (Mostafa and Miller, 2014). A review that looked at delirium cases after macrolides and examined case reports and FAERS database found that it happens in various age groups, including children (Pejčić, 2022).

Psychosis seems to be a rare side effect and is not always recognized by healthcare providers. A case report described a man who received prophylactic antibiotics, probably penicillin G benzathine, in the US military and developed paranoia and anxiety that progressed to schizophrenia. He faced a long fight to have his mental problems acknowledged as side effects. He first was dismissed with a diagnosis of posttraumatic stress disorder, although he had never been in combat or experienced any trauma from the military. When he mentioned to the physicians that he suspected antibiotics were the reason for his problems, he was dismissed as crazy (Ly and DeLisi, 2017).

A case report of a 65-year-old woman with no prior psychiatric history describes her presenting at the emergency department with unresponsive spells, agitation, and EEG abnormalities. Clinicians suspected autoimmune encephalitis, paraneoplastic limbic encephalitis, or a toxic–metabolic encephalopathy but found no positive results upon examination. Antiepileptic medication brought amelioration, but the woman showed hypomanic symptoms that improved over time. After 25 days at home, she again exhibited psychiatric symptoms and became paranoid. It was only during her second visit to the emergency department that her husband mentioned her treatment with clarithromycin for a *H. pylori* infection, leading to the recognition of the antibiotic as the cause of her problems. The antibiotic was discontinued; nonetheless, the woman presented at the emergency department with suspected focal seizures after 3 and 5 months (Whittam et al., 2024).

A search in the FAERS database revealed that 15 of the 23 investigated antibiotics were associated with an elevated odds ratio (OR = 1.67–9.48) for psychosis. The OR was calculated in comparison to minocycline, which was hypothesized to have protective properties and therefore a negligible risk. The antibiotics that had an increased odds were amoxicillin/clavulanate, ceftriaxone, sulfamethoxazole/trimethoprim, cephalexin, azithromycin, doxycycline, nitrofurantoin, erythromycin, cefuroxime, amoxicillin, cefepime, levofloxacin, metronidazole, ciprofloxacin, and clarithromycin. The antibiotics with the greatest increased odds were macrolides, fluoroquinolones, and metronidazole (Essali and Miller, 2020).

Common cognitive side effects of antibiotics are confusion, delirium, encephalopathy, and impaired concentration or attention. Risk factors include older age and renal impairment but are not limited to these. Occurrence of side effects can happen after 1–10 days, with resolution of symptoms after several days or weeks (Warstler and Bean, 2016).

### Neurological side effects

Neurotoxicity, including psychiatric side effects, is common among all classes of antibiotics. They affect the peripheral and central nervous system. A literature review by Madison K. Bangert and Rodrigo Hasbun examined the following classes: β-Lactams, Macrolides, Fluoroquinolones, Aminoglycosides, Polymyxins, Tetracyclines, Glycylcyclines, Sulfonamides, Glycopeptides, Lipoglycopeptides, Lipopeptides, Oxazolidinones, Lincosamides, Nitroimidazoles, Nitrofurans, and Antimycobacterials. The symptoms ranged from mild and transient manifestations like headaches and dizziness to severe encephalopathy, seizures, and irreversible polyneuropathy (Bangert and Hasbun, 2019).

In reviews of Grill & Maganti and of Rezaei et al. the following neurologic side effects were described: peripheral neuropathy, paraesthesia, encephalopathy, neuromuscular blockade, seizures, non-convulsive status epilepticus, tremors, asterixis, myoclonus, chorea, ataxia, cranial nerve toxicity, ototoxicity, optic nerve toxicity, intracranial hypotension, headaches, confusion, disorientation, psychological problems, insomnia, toxic psychosis (Grill and Maganti, 2011; Rezaei et al., 2018).

Shamik Bhattacharyya et al. summarized the neurological side effects of antibiotics and found central nervous system side effects such as seizures and encephalopathy (symptoms including confusion, somnolence, agitation, psychosis, and/or hallucinations), peripheral neuropathy (manifesting in sensory symptoms, weakness, and autonomic dysfunction), optic neuropathy, and antibiotic-induced exacerbation of Myasthenia Gravis (worsening of symptoms like weakness, dysarthria, dysphagia, and shortness of breath). Various classes of antibiotics were triggers of these side effects. Antibiotics associated with encephalopathy were metronidazole, fluoroquinolones, macrolides, and β-lactams, gentamicin, linezolid and trimethoprim-sulfamethoxazole. The incidence for epileptic seizures was low, ranging from 0.04% for cephalosporins to 0.1%–0.5% with fluoroquinolones. Antibiotics associated with peripheral neuropathy were metronidazole, linezolid, dapsone, chloramphenicol, chloroquine, ethambutol, fluoroquinolones, isoniazid, nitrofurantoin, and sulfasalazine. Optic neuropathy was associated with ethambutol, linezolid, ciprofloxacin, levofloxacin, chloramphenicol, metronidazole, sulfonamides, isoniazid, and streptomycin. Exacerbation of Myasthenia Gravis occurred with aminoglycosides, fluoroquinolones, macrolides, clindamycin, colistin, tetracyclines, ampicillin and imipenem. Older age, renal impairment and higher dosages were risk factors for the occurrence of symptoms (Bhattacharyya et al., 2014).

Among neurological side effects of antibiotics, central nervous system adverse reactions are an underrecognized phenomenon (Bhattacharyya et al., 2014; Khodakaram and Barmano, 2011).

There is an older Case report from 2009 of a 38-year-old woman who had 34 culture positive UTI and several suspected UTIS over a period of 7 years, almost all of them treated with Nitrofurantoin. She developed severe neuropathy. She got many diagnostic procedures, diagnoses and treatments. Intermittently she was also treated for suspected MS. Over a period of 3 years, she visited eight different physicians, and none recognized her symptoms as side effect, despite the patient’s disclosure of her chronic use of nitrofurantoin. An informal poll of obstetricians, gynaecologists and urologists at the medical centre by the authors of this case report did not reveal any individual who had knowledge of this neurotoxicity (Kammire and Donofrio, 2007).

A study that performed the largest case study to date found 10 cases of metronidazole induced neurotoxicity (MIN). Typical lesions found in MRI are those involving the cerebellum, brainstem, and corpus callosum. Symptoms found in MIN include ataxia, polyneuropathy, dysarthria, dysmetria, altered mental status, and seizures. Of the 10 cases, in 9 cases symptoms resolved after discontinuation of the medication. In one case, polyneuropathy did not resolve. MIN occurs more often with high cumulative dosages, but one case involved a patient who took metronidazole for under 1 week, suggesting that the occurrence of pathologies might not be dose dependent. An 8-year-old patient had received protective metronidazole treatment for several years due to transplant immunosuppression. As his symptoms consisting of falls started, metronidazole as the cause was not considered. The differential diagnosis included mass lesion, postinfectious ataxia, and vitamin deficiency (Patel et al., 2020).

A search of CNS side effects of cephalosporins in the French national vigilance database revealed 511 serious reports of CNS (neurological and psychiatric) side effects in a time frame of 30 years. Serious cases were defined as those requiring hospitalization, being life-threatening, or resulting in disability. Individuals were at elevated risk if they had concomitant CNS disease, were on other medications, were over 65 years old, or had renal impairment. The most frequently reported side effects were encephalopathy, convulsions, myoclonia, and psychiatric effects including confusional state and hallucinations. In 18% of cases, there was a fatal outcome (Lacroix et al., 2019).

Raoul Sutter et al. conducted a review on epileptic seizures, including articles from 1960 to 2013. Analysing 65 articles with 25,712 patients and 25 different antibiotics, they found that the association between antibiotics and seizures is low to very low. The risk was particularly increased for unsubstituted penicillins, fourth-generation cephalosporins, imipenem, and ciprofloxacin. High doses, concomitant brain lesions or known epilepsy, and renal impairment further increased the risk. Reducing the antibiotic dose led to the cessation of seizures in some cases. Most seizures are tonic-clonic, but seizures related to antibiotic drugs can be easily overlooked or misdiagnosed as delirium or encephalopathy when they are nonconvulsive (Sutter et al., 2015).

A case report of an elderly woman experiencing life-threatening seizures under nitrofurantoin demonstrates that epileptic seizures can occur with antibiotics not typically associated with this side effect, and this is not described in the drug’s prescribing information (compendium, 2024; Stuhec and Svab, 2012).

### Mechanisms of antibiotic neurotoxicity

Several mechanisms of neurotoxicity are discussed, one of them is mitochondrial toxicity.

Mitochondria are well known as the energy producing organelles in the cells that produce the energy rich molecule ATP by utilizing Glucose or fat from nutrition. But their functions go way beyond that. Mitochondria also play an important role in many other cellular events, for example, in calcium homeostasis, oxidative stress control and inflammation. The Endosymbiotic Theory suggests that mitochondria originated from alpha-proteobacteria that formed a symbiotic relationship with early eukaryotic cells (Margulis, 1975). Many antibiotics that target the bacterial mechanisms can also target and damage mitochondria and lead to cytotoxicity. (Suárez-Rivero et al., 2021).

A review of D’Achille and Morroni looked at the different mechanisms that antibiotics have on mitochondria and found the following (D’Achille and Morroni, 2023):

- Oxazolidinones: Mitochondrial protein synthesis reduction, reduction of adenosine triphosphate (ATP), Reduction of cytochrome c oxidase (COX) activity
- Aminoglycosides: Alteration of mitochondrial protein synthesis, mitochondrial membrane potential (MMP) loss, reactive oxygen species (ROS) production
- Tetracyclines: MMP loss, cytochrome c oxidase subunit I (COX-I) reduction
- Macrolides: Reduction of mitochondrial protein synthesis, mitochondrial swelling
- Phenicols: Cytochrome c release (this leads to apoptosis), reduction of ATP production
- Beta-lactams: Reduction of mitochondrial respiration, ROS production, apoptosis induction
- Colistin: Mitochondrial swelling, reduction of ATP
- Glycopeptides: MMP loss, ROS production
- Quinolones: MMP loss, cytochrome c release

Other mechanisms of antibiotic neurotoxicity that are discussed in a review by Grill & Maganti are (Grill and Maganti, 2011):

- Activation of NMDA receptors
- Axonal loss
- Inflammatory response
- Cytokine release
- Inhibition of GABA-A release
- Inhibition of GABA-A receptors
- Increased glutamate

Mitochondrial damage caused by antibiotics is well-documented in the literature. While inflammation is a known consequence of mitochondrial damage (Nesci et al., 2023), there is limited research exploring antibiotics as a potential cause of inflammation. In a different context than neurotoxicity, inflammation associated with antibiotics has been discussed. Kan et al. identify inflammation, alongside oxidative stress and kidney stone formation, as mechanisms through which vancomycin can induce acute kidney injury. Inflammation in this case is mediated by complement activation and T-cell activation. (Kan et al., 2022).

### Mitochondrial damage, release of DAMPs and neuroinflammation

Given that antibiotics are known to affect mitochondrial function, the following section explores hypothetical mechanisms through which they might contribute to neuroinflammatory processes implicated in neurodegenerative and psychiatric conditions.

When mitochondria are damaged, they can release mitochondrial damage-associated molecular patterns (mtDAMPs) into the extracellular space. These mtDAMPs—including mitochondrial DNA (mtDNA), mitochondrial transcription factor A (TFAM), and cardiolipin—are recognized by pattern recognition receptors on microglia, the resident immune cells of the central nervous system. This recognition triggers an innate immune response, leading to the release of pro-inflammatory cytokines and the initiation of neuroinflammation (Bajwa et al., 2019; Deus et al., 2022).

### Endoplasmatic reticulum stress

**FIGURE 1:**
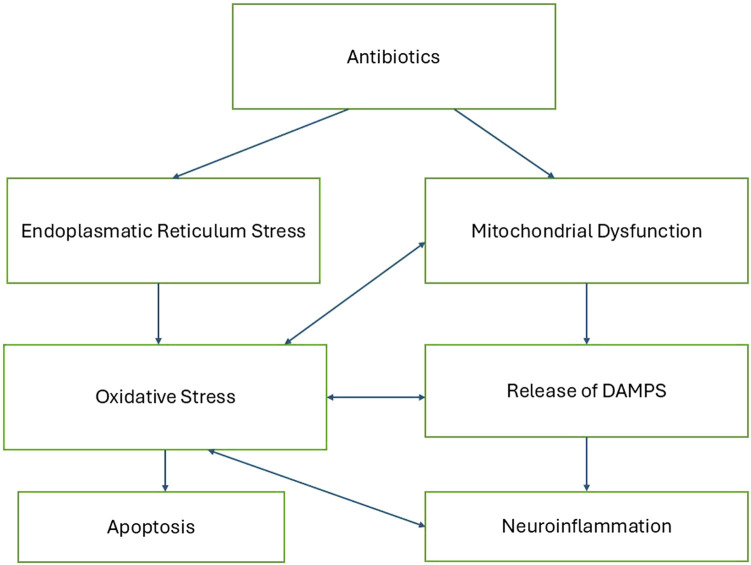
Proposed mechanistic pathway of antibiotic-induced neurotoxicity.

Antibiotic toxicity can be significantly influenced by endoplasmic reticulum (ER) stress, which plays a crucial role in the cellular response to various drugs. Antibiotics such as penicillinase-resistant antibiotics and macrolides have been shown to induce ER stress, leading to cellular damage and cytotoxicity. This stress is characterized by the accumulation of misfolded proteins and the activation of the unfolded protein response, which can result in oxidative stress and apoptosis if unresolved (Burban et al., 2018; Guillouzo and Guguen-Guillouzo, 2020). Figure 1 describes a proposed mechanistic pathway of antibiotic neurotoxicity that results in a vicious cycle.

### Mechanism of GABAA receptor antagonism

Another mechanism independent of mitochondria is GABAA Receptor toxicity. Ciprofloxacin has been shown to inhibit GABA-evoked currents in neuronal cells. Studies using the whole-cell patch-clamp technique on rat hippocampal neurons demonstrated that ciprofloxacin significantly reduces GABA-evoked currents, with the inhibitory effect being potentiated by the presence of non-steroidal anti-inflammatory drugs (NSAIDs) such as biphenyl acetic acid (BPAA) (Green and Halliwell, 1997; Halliwel et al., 1995; Halliwell et al., 1991).

### Microbiome changes and the brain-gut-axis

Hao et al., in their review, examine the relationship between antibiotic use, alterations in gut microbiota, and the development of depression. The authors highlight that disruptions to the intestinal flora play a significant role in the pathogenesis of depression. They discuss how antibiotic regimens can induce changes in gut microbiota composition, thereby affecting the brain-gut axis and potentially increasing the risk of depression. Mechanisms by which the microbiome can influence the brain include the regulation of nerve proliferation and the activation of glial cells, the production of neurotransmitters such as GABA and serotonin, or brain-derived neurotrophic factors. Additionally, the microbiome regulates short-chain fatty acids, which provide energy to the brain and act as signaling molecules. The disruption of the intestinal flora can also lead to increased permeability of the blood-brain barrier, with potential adverse effects on brain health (Hao et al., 2020). K. and T. Dinan, in their review, also discuss mechanisms by which microbes communicate with the brain *via* the vagus nerve and alter the production of tryptophan, the precursor of serotonin (Dinan and Dinan, 2022).

A systematic review and meta-analysis of 59 case-control studies identified a transdiagnostic pattern of gut microbiota perturbations across psychiatric disorders, including depression, bipolar disorder, schizophrenia, and anxiety. These conditions were consistently associated with a depletion of anti-inflammatory, butyrate-producing bacteria and an enrichment of pro-inflammatory bacteria (Nikolova et al., 2021). Similarly, a systematic review, meta-analysis, and meta-regression analyzing data from 2,091 patients with depression and 2,792 controls found comparable results. The study revealed that depression was characterized by an increase in pro-inflammatory bacteria and a significant reduction in anti-inflammatory butyrate-producing genera, underscoring a potential link between gut dysbiosis and systemic inflammation in mental health disorders (Gao et al., 2023).

### Neuroprotection of antibiotics?

Doxycycline has been shown to have neuroprotective properties in models of neurodegenerative diseases. In Alzheimer’s and Parkinson’s diseases, it protected against problematic protein formation, and in Parkinson and Multiple Sclerosis, it attenuated inflammation (Santa-Cecília et al., 2019). It has to be mentioned that doxycycline can be neurotoxic in rare cases leading to intracranial hypertension, neuromuscular blockade and cranial nerve toxicity (Grill and Maganti, 2011). There are also case reports of three young individuals without prior mental health problems that developed suicidality and even committed suicide in two cases under doxycycline for treatment of skin problems (Atigari et al., 2013). An epidemiologic study that wanted to find out if Doxycycline exposure in adolescence is associated with decreased risk of severe mental illness found a risk elevation of 95% for bipolar disorder (Upmark et al., 2021).

Also minocycline is discussed for its neuroprotective abilities (Plane et al., 2010) and is considered a potential therapeutic agent in psychiatric diseases (Dean et al., 2012). A review and meta-analysis found partly beneficial results like improvement of negative symptoms in schizophrenia but also mixed results in clinical trials for neurological and psychiatric disease (Panizzutti et al., 2023).

Nevertheless, in clinical settings, minocycline has been shown to have neurological side effects.

In a review on minocycline related side effects the following neurological side effects were listed: headache, dizziness, somnolence, tinnitus, vertigo, mood alterations, hypoesthesia, Idiopathic Intracranial Hypertension, paraesthesia, intracranial hypertension, impaired/decreased hearing, sedation, ataxia, vestibular reactions, convulsions (Martins et al., 2021).

A case report describes a 37-year-old woman with no prior psychiatric history who presented with periorificial dermatitis. She was prescribed minocycline and topical metronidazole cream. Two days after starting minocycline, she began experiencing depersonalization symptoms, a psychological condition where an individual feels disconnected or detached from their own body, thoughts, or emotions, as if observing themselves from outside. Nevertheless, she continued the medication for 3 weeks. After finding a similar experience online that described a reaction to minocycline, she decided to stop the medication. Her symptoms resolved after discontinuation, and a follow-up call from healthcare providers 1 month later revealed that the symptoms had not returned (Shamout et al., 2019).

### Epidemiologic studies on the use of antibiotics and risk of psychiatric diseases

Antibiotics are commonly prescribed in very young and vulnerable populations. A study examining one million births in Finland found that from birth to age two, 81% of children received at least one antibiotic prescription (Lavebratt et al., 2019).

Numerous epidemiological studies have explored the relationship between early life exposure to antibiotics, both *in utero* and during childhood to adolescence, and various psychiatric diseases. Generally, these studies have identified a moderately increased risk, necessitating a cautious and restrained use of these medications at the public health level.

A retrospective cohort study with a study population of over 800,000 individuals found that prescribing antibiotics in the first 2 years was associated with a 71% increased risk of psychotropic prescriptions in childhood, with a greater risk observed for prolonged or multiple antibiotic courses (Stark et al., 2022).

For children and adolescents aged 8–20 years following antibiotic treatment, there was a 27% increased risk for those who received broad-spectrum antibiotics to develop anxiety or depression. However, no increased risk was associated with the use of topical or narrow-spectrum antibiotics (Prichett et al., 2022).

Children with otitis media who were treated with broad-spectrum antibiotics exhibited an increased risk of autism, while otitis media alone also presented an independent risk (Wimberley et al., 2018).

A nationwide study in Denmark involving over one million individuals aged 1–18 years identified an 84% elevated risk for mental health diagnoses and a 42% elevated risk for psychotropic medication following hospitalization for infection. Antibiotic use was particularly associated with an increased risk for mental diseases. Conditions such as schizophrenia, OCD, personality and behaviour disorders, intellectual disability, autism, ADHD, conduct disorder, and tic disorder showed the highest risk following infection (Köhler-Forsberg et al., 2019).

Studies on antibiotic use during pregnancy and the early *postpartum* period show that in children aged 8–14, the risk associated with antibiotic use varies, indicating a 30%–120% increased likelihood of developing autism spectrum disorder or infantile autism, depending on the class of antibiotic used (HÓ et al., 2012).

Antibiotic exposure during the fetal period and the first two postnatal years was associated with a 10%–50% increased risk of developing various psychiatric disorders by age 18, including mood and anxiety disorders, sleep disorders, ADHD, and conduct disorders (Lavebratt et al., 2019).

A follow-up study of 526 children up to age 11, where 70% had antibiotic use in the first year of life, revealed more behavioural difficulties and symptoms of depression. Those treated with antibiotics had significantly lower intelligence test scores at ages 3.5, 7, and 11, lower reading ability scores at age 7, and higher behavioural difficulties at ages 3.5 and 7. By age 11, symptoms of ADHD and depression were more pronounced (Slykerman et al., 2017). The risk elevations for ADHD are mixed though because in a study population with over 600,000 children, antibiotic use during the first 2 years of life had no effect on ADHD (Axelsson et al., 2019).

One study found a positive effect of antibiotics on psychiatric symptoms. Antibiotic use for acne in a mentally healthy teenage population aged 15–18 years did not result in higher levels of depression, anxiety, or suicide. In fact, it led to an improved quality of life and reduced social anxiety and OCD (Erdoğan et al., 2019). Although another global study, comparing antibiotics to isotretinoin and involving over 75,000 participants in each group, found a higher risk of psychiatric disease in the antibiotic group than in the isotretinoin group (Kridin and Ludwig, 2023).

In an adult population, antibiotics also seem to lead to a small to moderate elevated risk for psychiatric diseases.

A systematic review analysed nine observational studies investigating the risk of depression. Studies conducted in the UK and Sweden, involving more than one million participants, demonstrated an increase in depression risk by at least 20%. Furthermore, the risk escalated with the number of treatment courses and the variety of agents used (Pouranayatihosseinabad et al., 2023).

A nested case-control study reported that the risk for depression and anxiety was increased by approximately 25% and 17%, respectively, and this risk increased with recurrent use of antibiotics. No increased risk for psychosis was observed (Lurie et al., 2015).

In a large-scale cohort study involving over one million individuals from the entire Danish population, researchers discovered a heightened risk of schizophrenia and affective disorders associated with antibiotic use. In contrast, the use of antiviral, antifungal and antiparasitic agents did not demonstrate a significant risk increase for these conditions. Notably, the risk was most pronounced with broad-spectrum antibiotics, which were associated with a 44% increased risk for schizophrenia and a 65% increased risk for affective disorders (Köhler et al., 2017).

In newly diagnosed cancer patients, antibiotic use during the year before the cancer diagnosis was associated with a 23% higher rate of psychiatric disorders such as first-onset psychosis, depression, anxiety, or stress-related disorders (Hu et al., 2022).

Armstrong et al. (2024) found a significant association between antibiotic use and fibromyalgia risk. Fibromyalgia is a chronic pain syndrome often associated with psychiatric comorbidities and is classified as a neurological disorder. The analysis was based on a large sample, comprising 44,674 individuals diagnosed with fibromyalgia and 133,513 matched controls. The findings revealed a strong association between prior antibiotic use and the likelihood of developing fibromyalgia. Compared to those with no antibiotic exposure, individuals who had received antibiotics faced a 127% higher risk of fibromyalgia. This risk increased further among those with the highest number of prescriptions, reaching nearly a 292% elevation, and was also markedly elevated in those with prolonged antibiotic use (228%). The antibiotics most closely linked to fibromyalgia onset included tetracyclines and metronidazole (Armstrong et al., 2024).

There was also a study that showed a risk reduction: one retrospective cohort study, utilizing Poland’s national database and examining 69 million patients after hospitalization, found a significant reduction in the risk of newly diagnosed mood disorders, anxiety, and stress-related disorders with antibiotic use. Additionally, the incidence of suicidality and psychotic disorders was observed to be lower in subjects over the age of 50 (Kerman et al., 2023).

### Epidemiologic studies on the use of antibiotics and risk of neurodegenerative diseases

The results for antibiotic use and risk of neurodegenerative diseases are mixed.

Tables 1 and 2 provide an overview of the various neuropsychiatric side effects of antibiotics and the different risk ratios for psychiatric or neurologic disorders reported in epidemiological studies.

**TABLE 1:** Neuropsychiatric effects of antibiotics.

Study reference	Study type	Antibiotic substance or class	Neuropsychiatric outcome
Tomé and Filipe (2011)	case reports and case series	fluoroquinolones	Abnormal dreams/nightmares, psychosis, agitation, aggression, anxiety, confusion, delirium, delusion, depression, euphoria, hallucinations, hostility, insomnia, irritability, mania, manic depression, mood swings, nervousness, panic attack, paranoia, personality disorder, sleep disorder, somatic delusion, suicidal ideation
Neufeld et al. (2017)	Case reports	Triple therapy: (proton pump inhibitor), clarithromycin and amoxicillin or metronidazole	Anxiety, delirium, dissociation, mania, psychosis
Abusafiyah and Soulen (2023)	FAERS database	Betalactams, Sulfomethaxole/Trimethoprim (SMX/TMP) Vancomycin, Gentamycin, Metronidazole	
Samyde et al. (2016)	Case reports (VigiBase)	Fluroquinolones	Suicide
Lambrichts et al. (2017)	Case reports	Clarithromycin, ofloxacin, ciprofloxacin, amoxicillin, erythromycin, cycloserine, ethambutol, ethionamide, isoniazide, pefloxacin	Mania
Mostafa and Miller (2014)	Case reports	Fluoroquinolones, SMX/TMP, penicillins	Psychosis
Pejčić (2022)	FAERS database	Macrolides	Delirium
Ly and DeLisi (2017)	Case report	Penicillin	Schizophrenia
Whittam et al. (2024)	Case report	Clarithromycin	Agitation, EEG abnormalities, paranoia, unrespronsive spells, psychosis
Essali and Miller (2020)	FAERS database	Amoxicillin/Clavunalate, ceftriaxone, SMX/TMP, cephalexin, azithromycin, doxcycycline, nitrofurantoin, erythromycin, cefuroxime, amoxicillin, cefepime, levofloxacin, metronidazole, ciprofloxacin, clarithromycin	Psychosis
Warstler and Bean (2016)	Case reviews/reports, retrospective cohort studies, retrospective chart reviews	Penicillins, cephalosporins, fluoroquinolones, macrolides	Confusion, delirium, encephalopathy
Bangert and Hasbun (2019) Grill and Maganti (2011) Rezaei et al. (2018)	Case reports, case series, review articles, animal models, clinical trials	All classes of antibiotics	Light headaches to severe encephalopathy, seizures and irreversible polyneuropathies, psychiatric problems
Bhattacharyya et al. (2014)	Review	Cephalosporins and fluoroquinolones Metronidazole, fluoroquinolones, macrolides, betalactams, gentamycin, linezolid, SMX/TMP Metronidazole, linezolide, dapsone, chloramphenicol, chloroquine, ethambutol Aminoglycosides, fluoroquinolones, macrolides, clindamycin, colistin, tetracycline, ampicillin, imipenem, paramixin Ethambutol, linezolid, ciprofloxacin, levofloxacin, chloramphenicol, metronidazole, sulfonamides, isozianid, streptomycin	Seizures encephalopathy Peripheral neuropathy Exacerbation of myasthenia gravis Optic neuropathy
Patel et al. (2020)	Case study	Metronidazole	Metronidazole induced neurotoxicity (MIN) with visible lesions in MRI
Lacroix et al. (2019)	French national vigilance database	Cephalosporins	Serious neurologic and psychiatric side effects
Sutter et al. (2015)	Case reports	Penicillins, cephalosporins, imipenem, ciprofloxacin	Seizures
Stuhec and Svab (2012)	Case report	Nitrofurantoine	Seizure
Kammire and Donofrio (2007)	Case report	Nitrofurantoine	Severe neuropathy
Atigari et al. (2013)	Case reports	Doxycycline	Suicidality/suicide
Shamout et al. (2019)	Case report	Minocycline	Depersonalization symptoms
Martins et al. (2021)	Review	Minocycline	headache, dizziness, somnolence, tinnitus, vertigo, mood alterations, hypoesthesia, Idiopathic Intracranial Hypertension, paraesthesia, intracranial hypertension, impaired/decreased hearing, sedation, ataxia, vestibular reactions, convulsions

**TABLE 2:** Epidemiologic studies: Antibiotics and risk of psychiatric or neurodegenerative disorder.

Study	Risk increase	Risk reduction
Antibiotic prescription in fist two life years and risk of psychotropic prescription in childhood (Stark et al., 2022)	71%	
Antibiotic treatment age 8–20 and risk to develop anxiety or depression Broad spectrum antibiotics Topical or narrow spectrum antibiotics (Prichett et al., 2022)	27% 0%	
Children treated with broad spectrum antibiotics and risk of autism (Wimberley et al., 2018)	30%	
Hospitalization for infection at age of 1–18 Risk for psychotropic medication prescription Risk for mental disease (Köhler et al., 2017)	42% 84%	
Antibiotic exposure during pregnancy and early *postpartum* period and risk of autism in children aged 8–14 (HÓ et al., 2012)	30%–120%	
Antibiotic exposure during fetal period and first 2 years of life and risk of psychiatric disease by the age of 18 (Lavebratt et al., 2019)	10%–50%	
Antibiotics in first 2 years of life and risk of ADHD (Axelsson et al., 2019)	0%	
Antibiotics in first year of life and risk of ADHD Executive function problems Metacognition function problems Impulsivity/Hyperactivity Emotional problems Anxiety (Slykerman et al., 2017)	70%–80% 60% 50% 50% 40% 30%	
Antibiotics and risk of depression in adults (Pouranayatihosseinabad et al., 2023)	20%	
Antibiotics and risk of depression Anxiety Psychosis in adults (Lurie et al., 2015)	25% 17% 0%	
Broad-spectrum antibiotics and Risk of Schizophrenia Affective disorders (Köhler et al., 2017)	44% 65%	
Antibiotics 1 year before cancer diagnosis and risk of psychiatric disorders (psychosis, depression or stress-related disorder) Hu et al. (2022)	23%	
Antibiotics and risk of fibromyalgia (Armstrong et al., 2024)	127%–292%	
Antibiotic exposure during hospitalization and risk of mood disorders Anxiety and stress-related disorders Suicidality in men Suicidality over 50 years Psychotic disorder over 50 years (Kerman et al., 2023)		6%–16% 7%–12% 7% 33% 17%
Antibiotic use and risk of dementia (Rakuša et al., 2023)	0%	
Antibiotic use and risk of dementia 1–30 days of treatment 31–90 days of treatment 91 days and longer (Kim et al., 2022)	9% 23% 44%	
Antibiotics and Risk of Multiple Sclerosis (Hosseinzadeh et al., 2023)	0%	
Antibiotics and Risk of Parkinson (Palacios et al., 2020)	0%	
Antibiotics and Risk of Parkinson Macrolides and Lincosamides (Mertsalmi et al., 2020)	Up to 42%	
Antibiotics and risk of ALS One prescription 2–3 prescriptions 4 or more prescriptions More than 2 beta-lactamase sensitive penicillins (Sun et al., 2019)	6% 13% 18% 28%	
Maternal infection treated with antibiotics during first trimester and risk of schizophrenia in infant (Sørensen et al., 2009)	153%	
Treated severe pneumonia and risk of Any dementia Alzheimer’s Vascular dementia Unspecified dementia (Chu et al., 2022)	183% 144% 315% 163%	

Three separate studies investigating the association between antibiotic use and the risk of dementia reported divergent findings. A nested case-control study utilizing German health claims data with over 35,000 dementia cases, after adjustment for comorbidities, bacterial infection and disease, observed no increased risk of dementia; indeed, a negative association was noted in certain subgroups of antibiotics (Rakuša et al., 2023). In contrast, a retrospective cohort study in South Korea, involving more than 300,000 participants, identified varying risks associated with different durations of antibiotic treatment. Specifically, the risk for overall dementia was 9% for 1–30 days of treatment, increased to 23% for 31–90 days, and reached 44% for durations of 91 days or longer (Kim et al., 2022). A further study examining a cohort of over 14,000 women from the Nurses’ Health Study II found that women who had taken antibiotics for at least 2 months during midlife exhibited small declines in cognitive scores on neuropsychological tests conducted 7 years later (Mehta et al., 2022).

A systematic review and meta-analysis aimed to reconcile the conflicting results regarding the association between antibiotic use and the risk of developing multiple sclerosis. This analysis included five studies, encompassing a total of over 470,000 participants. The authors concluded that there was only a non-significant association between antibiotic use and the incidence of multiple sclerosis, with an odds ratio of 1.01, corresponding to a 1% increase in risk. The individual studies analysed within the meta-analysis reported odds ratios ranging from 31% risk reduction to 52% risk increase (Hosseinzadeh et al., 2023).

The Nurses’ Health Study, which examined the association between antibiotic use and the risk of Parkinson’s disease in over 59,000 women, found no significant association. An important limitation acknowledged by the authors was the reliance on participants’ recall of antibiotic use from decades earlier (Palacios et al., 2020). In contrast, a nationwide case-control study in Finland involving over 13,000 Parkinson’s disease cases and more than 40,000 controls identified differences in risk among subgroups of antibiotics. The highest risk, with an increase of up to 42%, was observed for macrolides and lincosamides. A positive association was also noted for broad-spectrum antibiotics in general, as well as sulphonamides and trimethoprim (Mertsalmi et al., 2020).

Another study examining antibiotic consumption patterns across 30 European countries identified a specific risk elevation associated with narrow-spectrum penicillin, while broad-spectrum antibiotics generally showed a negative association. Particularly concerning was the positive association found between quinolones and Parkinson’s disease-attributed death rates (Ternák et al., 2022).

In a Swedish study involving over 2,400 amyotrophic lateral sclerosis (ALS) patients, a small but statistically significant association was found between antibiotic use and the risk of developing ALS. The risk was correlated with the number of antibiotic prescriptions received: a 6% increase in risk for one prescription, 13% for two to three prescriptions, and 18% for four or more prescriptions. Notably, the risk of ALS was particularly elevated, at 28%, with the administration of more than two prescriptions of beta-lactamase-sensitive penicillin (Sun et al., 2019).

### Bacterial infection and risk of psychiatric or neurological disease

Not only with antibiotics but also with bacterial infections themselves, the risk for neuropsychiatric disease is increased (Abdoli et al., 2020). Analyzing epidemiological studies is difficult because most infections are treated with antibiotics, leading to confounding. For example, a Danish study found an up to 153% times increased risk for developing schizophrenia when infants were exposed to maternal bacterial infection during the first trimester. However, bacterial infection was coded only when a medical diagnosis was made or treatment was conducted, making it difficult to discern from the risk increase by the pathogen itself (Sørensen et al., 2009).

The same with a cohort study that looked at the National Health Insurance Research Database in Taiwan to assess the risk of dementia following bacterial pneumonia. The risk increased by 183% for any dementia, 144% for Alzheimer’s Disease, 315% for Vascular Dementia, and 162% for unspecified dementia. The study participants were all severely ill and were treated (Chu et al., 2022).

There are efforts while looking at psychiatric symptoms to disentangle infections from antibiotic treatment as a cause. A study on pediatric patient with autoimmune neuropsychiatric disorders associated with streptococcal infections (PANDAS) did not find a connection between antibiotics and onset of disease. PANDAS are a subtype of acute-onset obsessive-compulsive disorder (OCD), believed to result from an autoimmune response to group A streptococcal infections (Sigra et al., 2018). Avis Chan and Jennifer Frankovich had been contacted from concerned parents and clinicians that suspected antibiotic side effects in children with PANDAS and they examined if OCD was a possible side effect of the antibiotic. They systematically reviewed electronic medical records of their PANDAS patients and looked at antibiotic exposure and development of psychiatric symptoms. Their study cohort included 88 patients. 60% of children had an infection within 12 weeks prior to the onset of symptoms of which only 14% had received antibiotics. 84% of children had no antibiotic exposure 6 months prior to the development of psychiatric symptoms (Chan and Frankovich, 2019).

### Rescue of antibiotic cell damage by antioxidants

#### N-Acetylcysteine

N-Acetylcysteine (NAC) is a medication that is widely used as antidote against paracetamol overdose and as mucolytic agent and is well tolerated. It is an effective antioxidant and has anti-inflammatory properties (Tenório et al., 2021). It is also studied as an enhancement of antibiotic efficacy (Petkova et al., 2023). Up to date it has been studied in animal models for its neuroprotective properties and in clinical trials for psychiatric disease (Bavarsad et al., 2014). There are several studies that show that NAC protects mammalian cells, and specifically brain cells from antibiotic damage:

Doxorubicin (DOX) is a cytostatic antibiotic used in cancer treatment (Johnson-Arbor and Dubey, 2024). Fifty rats were divided into four groups: a control group, a group that received only NAC, a group that received only (DOX), and a group that received both DOX and NAC. After 4 weeks, their brains were examined. The rats that received only DOX had higher mortality rates and showed more pronounced signs of weakness, as well as a decline in food and water consumption compared to the rats that were given both DOX and NAC. Rats treated with both DOX and NAC showed reduced inflammation (lower TNF-α levels), improved antioxidant status (higher GSH and glutathione peroxidase levels), and increased lipid peroxidation compared to those treated with DOX alone. Additionally, NAC pretreatment partially improved the histological condition of the brain, reducing the severity of neuronal shrinkage observed in the DOX-treated group. These findings suggest that NAC may offer protective benefits against DOX-induced neurotoxicity (Mohammed et al., 2019).

Human cell lines treated with clinically relevant doses of bactericidal antibiotics—quinolones, aminoglycosides, and β-lactams—showed mitochondrial dysfunction and overproduction of reactive oxygen species (ROS) with damaged cell components. In the same study, mice treated with the quinolone Ciprofloxacin exhibited elevated oxidative stress markers in the blood, oxidative tissue damage, and upregulated expression of key genes involved in antioxidant defense mechanisms. Use of bacteriostatic antibiotics in cell culture did not lead to those damages. All the negative effects of bactericidal antibiotics in cell culture and *in vivo* were alleviated by the administration of NAC. It was also found that NAC did not decrease the antimicrobial activity of the antibiotics (Kalghatgi et al., 2013).

Rats treated with clinically relevant dosages of the bacteriostatic antibiotic tetracycline were examined for effects on their testes. The treated rats exhibited decreased testicular weight, reduced epididymal sperm motility, lower percentages of live spermatozoa, decreased sperm count, and an increase in abnormal sperm morphology. Additionally, histopathologic changes were observed in the testicular tissue. Administration of Vitamin C and NAC significantly mitigated the toxic effects of tetracycline on sperm parameters and antioxidant marker enzymes, although the histopathologic changes remained unaffected (Farombi et al., 2008).

A study showed that clinically relevant concentrations, metronidazole, tigecycline, clindamycin, and azithromycin were found to induce apoptosis in human primary neuron cells. In contrast, ampicillin and sulfamethoxazole, even at high concentrations (up to 400 μg/mL), did not induce apoptosis in neuron cells. Specifically, tigecycline (40 μg/mL), clindamycin (200 μg/mL), and azithromycin (20 μg/mL) completely inhibited both basal mitochondrial respiration and maximal respiratory capacity in neuronal cells. Pretreatment with NAC completely negated the antibiotics’ effects of increasing intracellular ROS and mitochondrial superoxide. Moreover, NAC restored mitochondrial membrane potential, respiration, and ATP production to levels comparable to those in control cells (Xiao et al., 2019).

#### Neuroprotection by ketone bodies

In 1920s, the ketogenic diet was developed for the treatment of epilepsy (Wheless, 2008). Ketones (beta-hydroxybutyrate, acetone, and acetoacetate) are produced mainly by the liver (Bendridi et al., 2022) when an individual follows a ketogenic diet consisting of less than 20 g carbohydrates and more than 70% fat per day (Wheless, 2008).

The metabolism shifts to a state known as nutritional ketosis, characterized by blood levels of beta-hydroxybutyrate ranging from a minimum of 0.5 mg/dL to 3 mg/dL (Phinney and Volek, 2011). In this state, the brain transitions from primarily using glucose as an energy source to predominantly using ketones. This shift has many benefits for the brain: energy production from ketones is more efficient than from glucose (García-Rodríguez and Giménez-Cassina, 2021) and can bypass brain glucose hypometabolism (Myette-Côté et al., 2022). Furthermore, ketone bodies possess anti-inflammatory and antioxidant properties (Myette-Côté et al., 2022), stimulate mitochondrial biogenesis (Maalouf et al., 2009), and are beneficial for the gut microbiome (Ang et al., 2020).

The possibility of increasing ketones in the blood exists not only through dietary measures but also through the natural production of ketones by fasting. Additionally, there is the option of supplementing with exogenous ketones in the form of ketogenic fats like MCT oil or with ketone salts and ketone esters. They all lead to an increase in blood ketone levels (Poff et al., 2021).

With all these properties, ketones may potentially protect against the neurotoxicity of antibiotics. Especially with prolonged antibiotic administration, a ketogenic diet could have a protective effect, or the use of supplements in form of exogenous ketones could be considered during short-term antibiotic administration.

In a mouse model of kidney injury induced by cisplatin, pretreatment with the ketone beta-hydroxybutyrate was able to protect kidney tissue. This pretreatment could mitigate inflammation, oxidative stress, and tubular apoptosis (Kim et al., 2024).

MCT oils, that are converted into ketones in the liver, and ketogenic diet have already demonstrated beneficial effects in Alzheimer’s disease (Avgerinos et al., 2020; Bohnen et al., 2023), and the ketogenic diet has shown substantial benefits in mental disorders such as bipolar disorder, major depression, and schizophrenia (Danan et al., 2022; Sethi et al., 2024).

## Discussion

Chris Palmer MD, in his book “Brain Energy”, describes psychiatric and neurological disorders as metabolic disorders of the brain. He highlights that individuals with metabolic disorders of the body, such as diabetes and heart disease, are at a greater risk of developing psychiatric or neurological disorders and *vice versa*. Palmer identifies mitochondria as the connecting link in these conditions (Palmer, 2022).

Antibiotic toxicity is not only discussed in the context of neurological or psychiatric diseases but also in relation to their potential roles in other chronic diseases. Epidemiologic studies have shown an increased risk for diabetes type 2, obesity (Nuotio et al., 2022), cancer (Roderburg et al., 2023) and an increased risk for all-cause and cardio vascular mortality (Heianza et al., 2020).

Antibiotics can disrupt cellular functions, leading to histopathological changes. Specifically, fluoroquinolones, which are known for their neurotoxic effects and toxicity to multiple organs, have a recommendation for restricted use in the UK as a reserve antibiotic and carry a black box warning in the US due to acknowledged toxicity. These restrictions are supported by thousands of lawsuits from affected individuals since the antibiotics’ introduction in the 1980s (Drugwatch.com). It took many years for the official acknowledgment of the occurrence of serious damage, the FDA black box warning for permanent nerve damage was issued in 2013 (Centers for Disease Control and Prevention, 2023) and for mental health problems in 2018 (Commissioner O of the. FDA. FDA, 2020), the UK recommendation to use fluoroquinolones only as a reserve antibiotic in 2024 (*Fluoroquinolone Antibiotics*, n.d.-a.).

It is known that antibiotics can cause transient neurological or mental health problems, with most symptoms resolving upon discontinuation of the medication. However, less is known about potential long-term negative effects. It is known that fluoroquinolones can cause permanent damage, but the frequency of similar effects with other antibiotics requires further research. For fluoroquinolones, patients had trouble making their physicians believe that their psychiatric problems came from the antibiotic (Kaur et al., 2016). Similar reactions are expected to happen with other classes of antibiotics, contributing to the under-recognition of their toxicity. The possibility that cellular defects may appear with a delay and that this makes it difficult to attribute symptoms to antibiotics also must be taken into consideration.

The toxicity of antibiotics extends to other classes as well. Cases of antibiotic-related adverse effects are often underreported or unrecognized, raising concerns about the extent to which antibiotics contribute to chronic diseases. Toxicity is a rare effect, suggesting that certain individuals may be more susceptible. For instance, psychiatric diseases have been linked to genetic variants that predispose individuals to mitochondrial dysfunction (Li et al., 2021), (Brennand et al., 2015), (Triebelhorn et al., 2022), which may increase their vulnerability to antibiotic toxicity.

The review of Juan M. Suárez-Rivero et al. discusses various classes of antibiotics as possible therapeutics for neurodegenerative disease. It focuses on anti-inflammatory and mitochondrial enhancing properties of antibiotics, among others also fluoroquinolones as potential therapeutics are discussed (Suárez-Rivero et al., 2023).

However, the question remains whether antibiotics could be beneficial for some individuals while potentially toxic for others. For instance, those with a genetic predisposition to dysfunctional mitochondria might experience worsened conditions with the onset of neurological or psychiatric diseases. As reactions to antibiotics are idiosyncratic it is not possible to predict who will have serious side effects and the pros and contras of use of antibiotics should be carefully considered.

Antioxidants and ketones have been shown to protect cells from oxidative stress and mitochondrial damage. With all their properties, ketones may potentially protect against the neurotoxicity of antibiotics. Especially with prolonged antibiotic administration, antioxidants, a ketogenic diet could have a protective effect, or the use of supplements in form of exogenous ketones could be considered during short-term antibiotic administration.

Neurotoxicity as a side effect of antibiotics seems to be rare but can sometimes be a serious event, often underestimated and underrecognized. It occurs more frequently with prolonged use, in the elderly, and in individuals with impaired renal or liver function. However, healthy individuals can also be affected, sometimes after short durations of treatment and small dosages. Some promising studies show cellular protection by antioxidants, but more research, especially on humans, is needed to have conclusive results that justify the use of antioxidants as adjunct therapy with antibiotics.

The urgency of this topic is heightened by the growing problem of antibiotic resistance, which forces reliance on more toxic antibiotics. Vulnerable populations, such as the elderly and those with chronic conditions, are particularly at risk of adverse effects from these medications. Additionally, as antibiotics are among the most widely prescribed drugs, even rare side effects could impact a significant number of people. Addressing these issues is not only a matter of individual health but also a pressing public health concern.

Science must take proactive steps to better understand antibiotic-induced neurotoxicity and to develop protective interventions. Promising studies on antioxidants and ketones suggest potential pathways for reducing harm, but robust clinical trials are necessary to validate these findings. As the prevalence of antibiotic use and resistance grows, so does the urgency for research to inform safer prescribing practices and mitigate long-term harm to patients.

## Conclusion

Neurotoxicity as a side effect of antibiotics seems to be rare but can sometimes be a serious event, often underestimated and underrecognized. It occurs more frequently with prolonged use, in the elderly, and in individuals with impaired renal or liver function. However, healthy individuals can also be affected, sometimes after short durations of treatment and small dosages. As antibiotics are among the most prescribed medication numerous people could be affected. Some promising studies show cellular protection by antioxidants, but more research, especially on humans, is needed to have conclusive results that justify the use of antioxidants as adjunct therapy with antibiotics.
